# Report on First Iranian International Conference on Women’s Health Held June 14-15, 2012 in Shiraz, Iran

**Published:** 2013-03-06

**Authors:** Najmeh Maharlouei, Seyed Mehdi Ahmadi, Kamran B. Lankarani

**Affiliations:** Health Policy Research Center, Shiraz University of Medical Sciences, Shiraz, Iran

**Keywords:** Meeting Report, Women, Health, Conference

## Abstract

Women’s health is increasing identified as a global health priority. Women’s health is affected by many factors, such as the economy, environment, society, culture, religion, and biology. For this reason, the Health Policy Research Center, Shiraz, Iran decided to hold The First Iranian International Conference on Women’s Health. The aim of this conference was to provide up-to-date information on different aspects of women’s health, including healthy aging, non-communicable and communicable diseases, psycho-social aspects, health promotion, reproductive health, and nutrition. Finally, the attending specialists and experts provided recommendations to be put into practice which reinforced the recommendations for additional clinical preventive services for women, mobilizing health professionals within practice, education, and research to address the national health goals, encouraging the adoption of ongoing evidence-based prevention guidelines, gender-sensitive, and culturally appropriate, persuading all stakeholders to harmonize their endeavors on women’s health, changing the viewpoint to the women as a workforce alliance as with like men, along with considering the major role of women as the basis of the family, and improving the coverage, accessibility, and quality of women-oriented health services.

## Introduction

Women’s health is identified as a global health priority. Women’s health is affected by many factors, such as the economy, environment, society, culture, religion, and biology (1). In addition, biological changes during throughout their lives such as menstruation, menopause, sexual and reproductive aspects, as well as care-giving to children and elderly members of the family leads them to use more health services. Nonetheless, their having access to and utilization of appropriate health services is debatable (2).

There are also some other factors which are combined to form a lower quality of life and more health risks for women including their higher life expectancy compared to men in most countries, unequal access to health information and basic health support along with specific health hazards, such as physical and sexual assault, sexually transmitted diseases(STDs) (3).

In 2009, the Department of Gender, Women, and Health of the World Health Organization (WHO) officially announced ten facts regarding women’s health (4):

1. The epidemic in sub-Saharan Africa is increasingly female.
2. Tobacco use is rapidly rising among the younger women in developing countries.
3. Violence has serious health consequences for women.
4. Violence against women is widespread worldwide.
5. Even though early marriage is on the decline,100 million girls are estimated to marry before their 18th birthday over the next 10 years.
6. Most adolescent mothers live in developing countries.
7. Essentially all maternal deaths occur in developing countries.
8. When women earn an income, they are more likely than men to buy nets for their house holds to prevent malaria.
9. The burden of chronic obstructive pulmonary disease (COPD) is over 50% higher among women in comparison to men.
10. Women have a higher risk of becoming visually impaired than men.

It is clear that some facts may be different between developing and developed countries. Nonetheless, there are many commonalities in health challenges facing women worldwide. For instance, by improving perinatal care and reducing maternal mortality rates, women from developing countries will live longer and are less likely to suffer from poor health and premature mortality. In addition, the trend of changing mortality is similar to that of the developed countries; i.e., women have to live with chronic diseases for years (5).

These findings indicate that despite remarkable progress in the past decades, health organizations (national and international) have unsuccessfully fulfilled the health care needs of women at major courses of their lives, mainly during adolescence and older ages (6, 7).

Hence, WHO requests urgent action in dealing with non-governmental organizations (NGOs) and national health providers to ameliorate the health status as well as the quality of life of females of all age groups from teenagers to the elderly worldwide. As an urgent action toward women’s health is in need, more targeted and reliable researches are being conducted in this field (8).

Under these circumstances, the Health Policy Research Center affiliated with Shiraz University of Medical Sciences, Shiraz, Iran decided to hold The First Iranian International Conference on Women’s Health on June 14-15, 2012, at Sina and Sadra Conventional Center in Shiraz, Iran.

This conference was held in collaboration with the University of California, Los Angeles (UCLA, Los Angeles, CA, USA), Johns Hopkins Bloomberg School of Public Health (Baltimore, MD, USA), Ryerson University (Toronto, Canada), Leyden Academy on Vitality and Ageing (Leiden, Netherlands), United Nations Population Fund (UNFPA, Tehran, Iran), Iran Academy of Medical Sciences (Tehran, Iran), Centre for Women and Family in the President Institution (Tehran, Iran), Iran Society of Midwifery (Tehran, Iran), Association of Food and Nutritional Health Supporter of Iran (Tehran, Iran), Iran Society of Gastroenterologists and Hepatologists (Tehran, Iran), Iran Association of Clinical Laboratory Doctors (Tehran, Iran), Iran Society of Endocrinology (Tehran, Iran), Iran Obesity Society (Tehran, Iran), Breast Feeding Promotion Society (Tehran, Iran), and Iran Society for Reproductive Medicine (Tehran, Iran). This conference aimed to provide up-todate, reliable information and research in these seven categories: women’s healthy aging, noncommunicable diseases, communicable diseases, psycho-social aspects of women’s health,health promotion, reproductive health and nutrition in pregnancy, breast feeding and menopause.

Overall, more than 900 national and international specialists, experts, and critics form various majors and subspecialties participated in this conference. During the two days of the seminar, experts stated their findings and experiences through oral and poster presentations. Panel discussions were also organized which provided an appropriate atmosphere for sharing and challenging hot topics in different fields of women’s health. A total of 865 abstracts in different subjects of women’s health were sent to the seminar scientificsecretariat, 350 of which were assigned for poster presentations. There were also 10 oral presentations.

Topics of the selected abstract for oral and poster presentation sranged from pure medical surveys to national and international health promotion projects as well as psychosocial and longterm aspects of women’s health. The main topics presented (oral or poster) were:

- Women’s health; what are the real challenges?
- Domestic violence
- Profile of intimate partner violence in women visiting an inner-city emergency department
- Psychological aspect of menopause
- Mental health status of women whose husbands have had sexual relations with other women
- Tension headache; disease burden in women
- Women in the mirror of beauty and glory
- How to prevent women’s osteoporosis in particular?
- How to have the least cardiovascular events in post-menopausal period?
- Quality of life in patients with endometriosis
- Vulvo-vaginal disorders
- Prevention of cancer of the cervix uteri
- Breast cancer; challenges and policies along with epidemiology
- Well-being into old age, life expectancy, and good health care for older women
- Health status of female refugees
- Dietary considerations to optimize healthy aging in postmenopausal women
- Nutritional needs of pregnant and lactating women
- How to promote breast feeding
- Controversies on elective cesarean section
- Maternal and neonatal outcome of cesarean section versus vaginal delivery
- Assessing environmental causes of birth defects through developmental toxicity experiments using accurate statistical models
- Epidemiology of Infertility

During the conference, Ms.Maria A.E. van der Waal who is the director at Leyden Academy on Vitality and Ageing and the Director at the International Longevity Centre of the Netherlands stated that human ageing has become one of the biggest challenges worldwide. The average life expectancy has increased in virtually all developed countries and women live longer than men. Life expectancy at the age of 65 in particular reflects the outcomes of the socio-medical systems to prevent and cope with chronic, ageassociated diseases. Nowadays, at the age of 65, life expectancy in the Netherlands is three years shorter for women and two years shorter for men compared to Japan, which has achieved the highest life expectancy in the world over the last two decades. However, if one compares this to the 1950s, these results would be reversed. In the industrialized countries, women live longer and face a longer burden of disease, meaning that women spend more years in poor health. The more years spent with a disease is caused by increased case detection, early diagnoses, and increased medical treatment of the risk factors. Future challenge is to limit the number of years with disabilities and diseases at the end of life. A good healthcare system will contribute to improving health and will decrease the number of years the people suffer from chronic diseases. This will most likely result in a further increase of the life expectancy.

Dr. Batool Ahmadi who is an Assistant Professor of Health Economics and Management Sciences at Tehran University of Medical Sciences, Tehran, Iran expressed the challenges of advancing women’s health in Iran. She elucidated that serious changes in women’s life at home, society, and work place have created new challenges for the country’s health sector, which requires deeper understanding of the concepts of women’s health in our country. As the fertility rate has sharply decreased in Iran, women’s health concepts and needs have increasingly included problems beyond reproduction and should include a more comprehensive and holistic view and approaches of women’s health experiences in their life span. Moreover, curative and preventive strategies should address the women’s most important difficulties which affect their health and, at the same time, resource allocation should be based on the women’s health priorities.

At the end of the conference, a final manifesto of the congress was declared. The main subjects were reinforcing the recommendations for additional clinical preventive services for women, mobilizing health professionals within practice, education, and research to address national health goals, encouraging the adoption of ongoing prevention guidelines which are evidencebased, gender-sensitive, and culturally appropriate, persuading all stakeholders (including academics, healthcare organizations, civil society, the private sector, donors, and healthcare workers) to harmonize their endeavors on women’s health, changing the viewpoint to the women as a workforce alliance like men along with considering the major roles of the women as the basis of the family, establishing elementary patterns for investigating women’s health policies, improving the coverage, accessibility, and quality of women-oriented health services, knowledge building and translation about women’s health needs, empowering psycho-social care of pregnant and postpartum women, and updating and expanding reproductive and sex health services for women in accordance with the newly-developed approach of higher level supports beyond maternity and reproduction.

The closing ceremony was accompanied by awarding the best presentations (a 30,000,000 Iran Rials or approximately 1400 USD research grant for each of the five winners). All participants were invited to attend the second annual international congress which will be held in 2013.

Finally, women’s health matters to women, their children and families, their communities, and the society as a whole. Accordingly, interventions aimed at changing personal behavior, such as sexual behaviors, tobacco and alcohol use, nutritional habits, and physical activity, or changing health care organizations and sectors; for example, guaranteeing access to medications and encouraging community-based care may remarkably reduce morbidity and mortality. Extensive strategies, such as poverty reduction, increased access to literacy training and education, and providing opportunities for women to participate in economic and social activities will also contribute to meeting the millennium development goals and making abiding achievements in women’s health.
